# Clinical Evaluation of a New Surgical Augmentation Technique for Transarticular Atlantoaxial Fixation for Treatment of Atlantoaxial Instability

**DOI:** 10.3390/ani13111780

**Published:** 2023-05-26

**Authors:** Franck Forterre, Ligita Zorgevica-Pockevica, Christina Precht, Kati Haenssgen, Veronika Stein, Pia Düver

**Affiliations:** 1 Division of Small Animal Surgery, Department of Clinical Veterinary Medicine, Vetuisse Faculty, University of Bern, 3012 Bern, Switzerland; ligita.zorgevica@unibe.ch; 2 Division of Clinical Radiology, Department of Clinical Veterinary Medicine, Vetsuisse Faculty, University of Bern, 3012 Bern, Switzerland; christina.precht@unibe.ch; 3 Division of Veterinary Anatomy, Vetsuisse Faculty, University of Bern, 3012 Bern, Switzerland; kati.haenssgen@unibe.ch; 4 Division of Clinical Neurology, Department of Clinical Veterinary Medicine, Vetsuisse Faculty, University of Bern, 3012 Bern, Switzerland; veronika.stein@unibe.ch

**Keywords:** atlantoaxial instability, toy breed dog, surgical augmentation technique, surgical stabilization

## Abstract

**Simple Summary:**

Instability between the two uppermost vertebrae (atlantoaxial instability) is a common problem, especially in toy breed dogs, resulting in a variety of neurologic symptoms. In most cases, surgical stabilization is indicated, but all of these procedures have some degree of complication, one of which is recurrent instability due to implant failure/loosening. To reduce this problem, a new augmentation technique involving additional wire/suture fixation through a bone tunnel in the second vertebra was evaluated, and the long-term clinical outcome was analyzed. Ten of the eleven dogs enrolled in the study had good to excellent clinical outcomes with no neurological symptoms. One dog died due to postoperative complications. In conclusion, the augmentation technique may be a valuable and technically feasible addition to the surgical stabilization of atlantoaxial instability in toy breed dogs, hopefully reducing the rate of recurrent postoperative instability.

**Abstract:**

The feasibility of a newly developed augmentation of ventral fixation technique for surgical stabilization of atlantoaxial instability was clinically evaluated in a cohort of eleven dogs, and long-term clinical outcomes were retrospectively analyzed. The new technique combines wire/suture fixation through a transverse hole in the axis anchored by two screws placed in the alae atlantis or at the cranial end of plates used to bridge the atlantoaxial joint ventrally. A previous biomechanical study demonstrated good stability of this technique during shear loading, comparable to the stability achieved with other standard techniques. Ten dogs improved clinically after surgery and returned to a normal life within 3–6 months of surgery. One dog developed aphonia, dysphagia, and died of aspiration pneumonia three days after surgery. The augmentation of conventional ventral atlantoaxial fixation with the transverse bony corridor of the proximal axis body may be a valuable way to enhance stabilization of the atlantoaxial joint.

## 1. Introduction

Atlantoaxial instability (AAI) is a common disorder of the cranial cervical spine that can result in compressive cervical myelopathy, particularly in small and toy breed dogs [1]. Affected dogs are mostly young animals due to the congenital patholophysiology of the disease, presenting with clinical signs ranging from pain and ataxia to tetraplegia [1,2,3,4]. Traumatic pathology is also possible [5]. Various treatment modalities have been described. These range from conservative treatment to various surgical techniques [2,3,4,6]. In many cases, surgical treatment is indicated because it allows alignment and stabilization of the spinal segment. Surgical stabilization techniques include dorsal and ventral fixation, with dorsal fixation including wiring, cross pins, nuchal ligament technique, and metallic retractors [1,2]. Ventral fixation consists of transarticular lag screws, pinning with and without polymethylmethacrylate (PMMA), screw fixation with PMMA, and plating [1,2,3,7,8,9,10,11,12]. Biomechanical studies have shown that different surgical stabilization techniques provide an improvement in AAI, with the advantage of ventral stabilization techniques being less prone to failure over time [3,13]. Despite the initial improved stability, all described stabilization techniques face different difficulties. The atlantoaxial region is a challenging anatomic site, and affected dogs are typically immature toy breeds dogs with very fragile and fine bony structures [14,15]. Implant loosening or failure is a common complication and can lead to recurrent instability and worsening of clinical signs [1,3,8,10,11]. Therefore, it is essential to achieve better bone purchase of the implants to reduce the risk of implant failure. Most commonly, implants are anchored in a ventrodorsal direction within the thin body of the axis (thickness of about 2–3 mm in toy breeds). However, anatomically, the axis is wider than it is tall. Using the width of the axis (thickness of about 8–9 mm in toy breeds) to secure the implant in place could, therefore, allow for better bone retention of implants. In a previous biomechanical study, we evaluated the stability of this new ventral augmentation technique in conjunction with a bilateral transarticular pin fixation technique [13]. In this previous study, the augmentation technique combined fixation with two k-wires driven across the atlantoaxial joint and wire fixation through a transverse hole in the axis fixed around two screws placed in the alae atlantis. The biomechanical study demonstrated good stability of this technique, which was comparable to other techniques, during shear loading tests [13]. 

The aims of our study were: (a) to evaluate the anatomical and clinical feasibility of the new technique of augmentation transarticular pin fixation, and (b) to evaluate the long-term clinical outcome of patients treated with the new technique. We hypothesized that (a) the surgical approach and placement of the additional wire would be feasible without major risks, and (b) the clinical outcome would be good to excellent with delayed or prevented implant loosening.

## 2. Materials and Methods

The study was a follow-up to the biomechanical study that was recently performed evaluating this augmentation method [13]. Transverse drilling had to be performed transcutaneously. We first performed a preliminary anatomical study to confirm that transcutaneous drilling could be performed without compromising an important anatomical structure. Therefore, a lateral approach was made to the vertebral body of the axis in a Chihuahua cadaver, and the area lateral to the body of the axis was anatomically dissected free. This allowed visualization of all structures in close proximity to the transcutaneous hole. 

The second part of the study evaluated the long-term (more than 6 months) clinical outcome following the use of the augmentation technique in cases of AAI.

### 2.1. Anatomical Study

**Figure 1:**
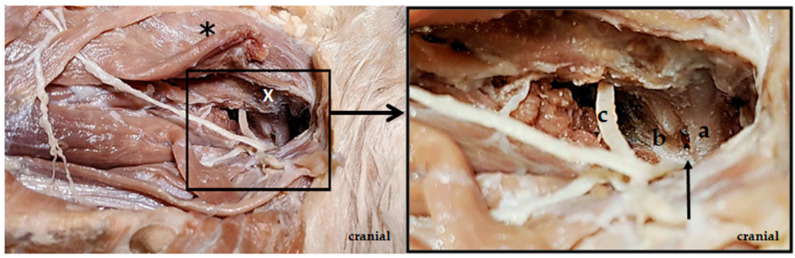
Cadaver dissection of the cervical spine of a dog. Lateral approach. The cleidocervical muscle fibers are split, and the omotransversarius muscle is dissected at the level of the alae atlantis (white x). The atlas (a), the axis (b), the joint space between atlas and axis (arrow) and the ventral branch of the C2 nerve (c) are clearly visible after the omotransversarius muscle is dorsally retracted (∗).

The skin of the cadaver was first opened, and the cutaneous muscle was cut. Then, the ventral nerve branch of C2 was exposed, and the omotransversarius muscle was dissected free. Along the ventral nerve branch of C2, the vertebral bodies of the atlas and axis were then advanced, and the area lateral to the vertebral bodies, as well as the main trunk of C2, and its branches were extensively dissected free. The second spinal nerve (C2) exits the spinal canal through the intervertebral foramen between the atlas and the axis in the area of the incisura vertebralis cranialis of the axis. At the exit from the spinal canal, the main trunk of the C2 spinal nerve, subsequently, separates into a dorsal and a ventral branch [16]. The ventral nerve branch of C2, as well as the axis and atlas, were clearly visible on the cadaver (Figure 1). The axis body was visibly located further ventrally than the ventral branch of C2. As the drill wire was inserted horizontally into the axillary body, the transcutaneous drilling occurred further ventrally than the ventral branch of the C2 runs. Accordingly, the risk of nerve damage from transcutaneous drilling was low. The vertebral artery runs along the side of the vertebral body [17]. This could be pushed ventrally with an arterial clamp so that the transverse drilling could be performed through the axillary body dorsal to the artery. The risk of injury to the artery is thus low. 

### 2.2. Case Selection

Dogs with confirmed evidence of AAI (traumatic or congenital) by CT or MRI and by surgical verification of displacement of the axis and compression of the spinal cord were included. They were treated by using the augmentation technique in association with a conventional stabilization technique (transarticular screws or pins, bilateral ventral plates bridging the atlantoaxial joint) via a ventral standard approach. Excluded were dogs that were treated without using the augmentation technique for AAI or dogs without any clinical follow-up. The medical records included breed, age, gender, body weight at surgery, cause of instability or type of fracture, clinical and neurological exams, and time of clinical onset and admission. Complications and early neurological state were recorded. A 6-month follow-up neurologic examination was available in all cases. Most commonly, this examination was performed by the referring veterinarian.

The following modified grading scale for cervical pathologies was applied: grade 0: no abnormal neurologic findings; grade 1: cervical hyperesthesia; grade 2: mild pelvic limb ataxia or paresis; grade 3: moderate pelvic limb ataxia or paresis; grade 4: marked pelvic limb ataxia with thoracic limb involvement; and grade 5: tetraparesis or inability to stand or walk without assistance [18,19].

### 2.3. Anesthesia

Anesthesia protocols were selected by the responsible supervising anesthesiologist (Dipl. ECVAA) according to the patient’s needs. Cefazolin (22 mg/kg IV) was administered half an hour before induction and continued every 1.5 h during surgery.

### 2.4. Diagnostic Imaging

#### 2.4.1. Radiographs

**Figure 2:**
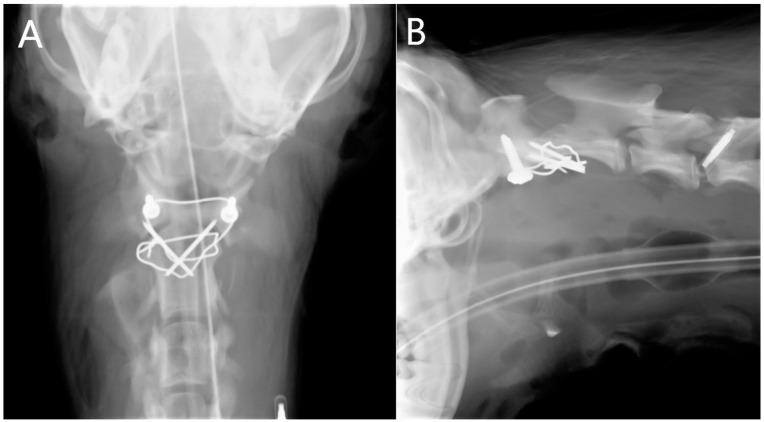
Ventrodorsal (**A**) and laterolateral (**B**) radiographs of the atlantoaxial region directly after surgical repositioning and transarticular atlantoaxial fixation. A wire fixation was used for augmentation.

Radiographs of the atlantoaxial region were generally performed by the referring veterinarian and were available at admission in the clinic. In some patients, post-operative ventrodorsal and laterolateral radiographs of the atlantoaxial region were taken (Figure 2).

#### 2.4.2. Computed Tomography

**Figure 3:**
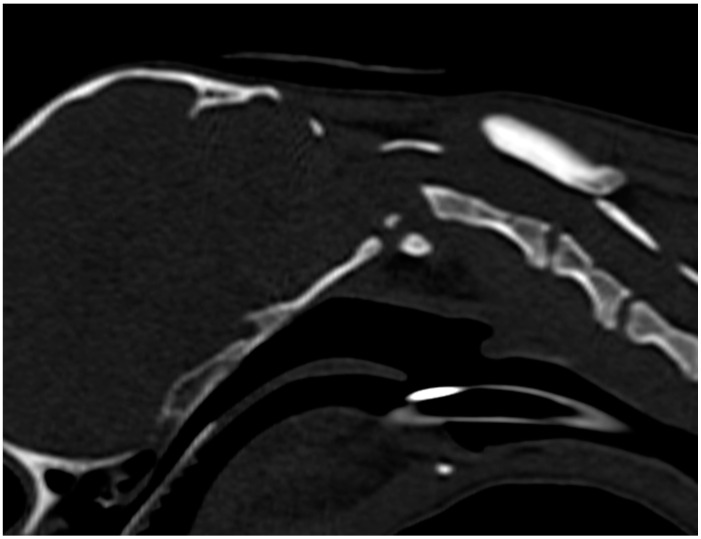
Sagittally reconstructed pre-operative CT image of the atlantoaxial region of the same case as in Figure 2, illustrating atlantoaxial instability with severe dorsal dislocation of the axis alongside with a hypoplastic and fragmented dens axis and occipital dysplasia.

**Figure 4:**
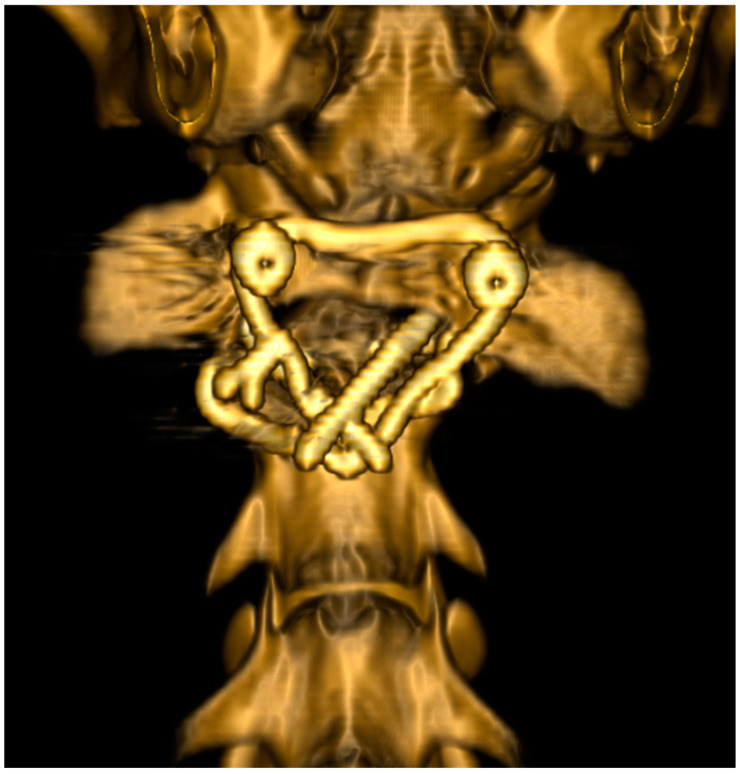
Three-dimensional rendered CT images of the atlantoaxial region of the same case as in Figure 2 and Figure 3 directly after surgical repositioning and transarticular atlantoaxial fixation. The augmentation wire was cranially positioned around the heads of the screws, which were fixed in the atlas.

CT of the head and cervical spine was performed pre-operatively (Figure 3) and post-operatively (Figure 4) to control reposition and fixation. Anesthetized dogs were positioned in dorsal recumbency with the head and neck in a neutral position and the forelimbs pulled caudally and fixed parallel to the thoracic wall. A sixteen-slice detector spiral CT (Philips Brilliance CT 16-slice scanner^®^, Philips AG Healthcare, Zürich, Switzerland) was used with a tube voltage of 120 kVp, a tube current of 195 mAs, a slice thickness of 1 mm, and an increment of 0.5 mm. Multiplanar reconstruction and volume rendering were used for assessment of the images. 

### 2.5. Magnetic Resonance Imaging

MRI examination was performed directly after CT in two cases preoperatively using an MRI unit with 1 Tesla field strength (Philips High Field Open^®^, Philips AG Healthcare, Zürich, Switzerland). The dogs were positioned in dorsal recumbency with a knee coil, including the head and cervical spine. Examinations included a sagittal and transverse T2-weighted fast spin echo (FSE) and a dorsal T1-weighted high-resolution gradient echo (FE 3D MPR). Additional sequences were performed when considered necessary. The slice thickness ranged from 1 to 2.5 mm in high resolution and 3–3.5 mm in the standard sequences. The atlantoaxial regions were assessed for signs of congenital abnormalities, instability, fracture, and spinal cord damage. The severity of spinal cord damage was estimated subjectively by evaluation of the amount of compression, deformation, displacement, and signal intensity changes within the spinal cord in the sagittal and transverse images. 

### 2.6. Surgical Technique

An area from the intermandibular space to the caudal cervical region, including the shoulder joints, was aseptically prepared, including the right lateral aspect of the neck. Animals were placed in dorsal recumbency, with the neck slightly hyperextended over a padded sandbag. The forelimbs were secured in a caudal position, and the head was taped to the table. 

**Figure 5:**
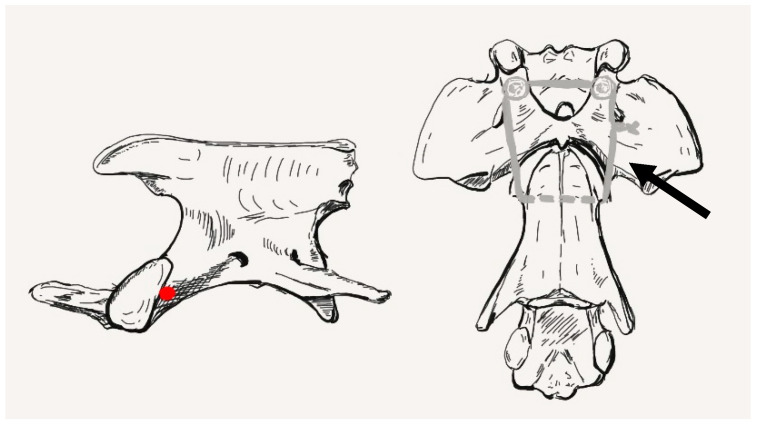
The red dot shows the location for the bone tunnel drilled into the axis, directly caudal to the articular surface. The ventral view shows the position of the wire/suture augmentation (arrow).

A standard ventral midline paramedian approach was made to the ventral aspects of C1 through C3 [20]. Surgical dissection was continued rostrally to expose the atlantooccipital joint. Periosteal dissection of the longus colli and rectus capitis ventralis muscles was performed along the midline, partially separating them from their bony attachments, followed by their lateral retraction. This exposed the median cervical region from the occipital bone to the cranial part of C3. Bleeding from the muscles was controlled with bipolar electrocautery before continuing the procedure. Using a previously described indirect reduction technique with 2 mini-Gelpi retractors, C1 and C2 were anatomically ligned [21]. The articular cartilage of the atlas and axis was removed using a pneumatic drill (Minos, 3M, Neuss, Germany) and a 2 mm burr. The subchondral bone was exposed to allow fusion of the atlantoaxial joint. The surgical wound was irrigated with lactated ringer’s solution. To augment the planned atlantoaxial stabilization technique, an additional wire/suture was pre-positioned to later stabilize the atlas and axis. A small skin incision was made on the right lateral aspect of the neck at the level of the cranial end of C2. A 1.1 mm drill guide was then inserted under visualization into the right cranial lateral aspect of the body of C2. The lateral muscles were gently deflected dorsally to allow visualization of the tip of the drill guide. A transverse hole was then made in the axis just posterior to the cranial articular surfaces (Figure 5). 

With the drill guide in place, the drill bit was removed, and a 0.5 mm wire or polypropylene filament (metric scale 4) was pulled through the hole until it merged 2–3 mm on the left side of the axis body. The tip of the wire on the left side was then clamped with forceps and pulled toward the ventral atlantoaxial region. The right end of the wire was similarly pulled through the skin toward the ventral atlantoaxial region. Prior to stabilization, a cancellous bone graft harvested from the craniodorsal region of the proximal right humerus or a bone morphogenetic carbon (BMC) implant was placed around and within the joint space. The standard technique for stabilization was then carried out. In cases where bilateral transarticular screw or pin fixation was chosen, two screws or pins were positioned ventrally into both atlas wings, and the ends of the 0.7 mm wire (e.g., polypropylene suture) were then twisted around the screw heads/pin. In cases of bilateral atlantoaxial plating, the wire ends were secured around the proximal edge of the plate or around the proximal screw within the plate. 

Finally, the bellies of the sternohyoid muscles and the subdermal fat tissue were apposed with 1.5 metric polydioxanone, the subcutaneous tissues with 1.5 metric polydioxanone, and the skin with a 1.5 metric polypropylene suture. 

### 2.7. Post Operative Care

The postoperative analgesia protocol consisted of fentanyl (5–10 mcg/kg/h IV) for 24 h followed by buprenorphine (Temgesic, Essex, Munich, Germany, 0.015 mg/kg SC or IV q8h) for 3 days. In addition, carprofen (Rimadyl, Pfizer, Berlin, Germany, 4 mg/kg PO q24h) was administered for 7 days and gabapentin (GabaLiquid GeriSan, Infectopharm Arzneimittel und Consilium GmbH, Heppenheim, Germany, 5–10 mg/kg PO q8h) for 3 weeks. Pain therapy was adjusted daily, according to the patient’s clinical status. A padded neck bandage extending from the lateral canthus of the eye to the midportion of the ribcage was applied for 4 weeks if tolerated. Daily neurological examinations were performed during hospitalization. Physiotherapy was started on the first postoperative day. Long-term follow-up (6 months) was obtained by sequential re-examination by the referring veterinarian or by telephone inquiry.

## 3. Results

Eleven dogs were enrolled in the study (for details see Table 1). Eight dogs were toy breeds (three Chihuahuas, two Miniature Spitz, one Havanese, one Yorkshire Terrier, and one Bolonka Zwetna), and three dogs were medium breeds (one Border Collie and two mixed breeds). In contrast to the small-breed dogs, which presented with a typical history of AAI without a major traumatic event, the medium-breed dogs were referred after a traumatic injury with atlantoaxial fractures associated with AAI. All dogs were between 3 and 16 months of age at presentation. Eight dogs were female, and three were male. Body weight ranged from 900 g (Chihuahua) to 11 kg (mixed-breed dog).

The elapsed time from onset of clinical signs to presentation to our clinic ranged from 3 days to 3 weeks. On neurological examination, the neurological location of the lesion was C1–C5 in all cases. One mixed-breed dog was tetraplegic with preserved deep pain sensation, two dogs were non-ambulatory tetraparetic, seven dogs were ambulatory tetraparetic, and one dog had only persistent cervical pain without neurological deficits.

The diagnosis of AAI was confirmed by CT scan in all cases. In nine cases, MRIs of the C1–C5 region were performed in addition to CT. Fractures of the atlas or axis were found in association with AAI in three cases. Late imaging follow-up at more than 6 months was performed in two cases and showed good reduction and stabilization of the AAI.

The augmentation technique was performed in all cases. A 0.5 mm wire was used in six cases and a 4 metric polypropylene filament in five cases. The technique was used in conjunction with transarticular pins in three cases, transarticular screws in two cases, and bilateral atlantoaxial plates in six cases. Technically, the correct placement of the wire was quite challenging in the first few cases. With time and the replacement of the wire with polypropylene, suture placement became easier. Postoperative dysphagia and respiratory dysfunction were observed in the first three cases treated with this technique. All three dogs were placed on oxygen supplementation and an esophageal feeding tube. One dog continued to deteriorate, despite supportive care, and died after 3 days. The other two dogs recovered rapidly and were discharged home after 5 and 7 days, respectively. No other short- or long-term complications related to the technique were observed. The remaining seven dogs showed no dysphagia or respiratory problems. 

Eight dogs were ambulatory at the time of discharge from the clinic, four were mildly tetraparetic (mean 5 days after surgery), and two dogs were tetraparetic non-ambulatory. Both recovered good locomotor function within 4 months of surgery. The mean postoperative follow-up time for the dogs in this study was 5–7 months for neurological follow-up by the private veterinarian and 6–13 months by telephone follow-up. One mixed-breed dog developed a thoracolumbar disc extrusion 8 months after surgery. The remaining patients all had uneventful follow-ups and showed normal locomotor function. 

## 4. Discussion

The addition of the augmentation technique to standard ventral fixation procedures was feasible during surgery, confirming our first hypothesis (hypothesis a). A learning and developmental curve was observed with severe complications occurring in the first three patients treated with the technique. With the exception of one dog that died from complications, all dogs improved neurologically postoperatively and returned to a normal life. As follow-up imaging was performed in only two dogs, the lack of late diagnostic imaging controls did not allow us to confidently state that the augmentation technique improved long-term stability by delaying implant loosening in all patients, but the long-term clinical outcome was good to excellent in all but one patient (hypothesis b).

Due to the challenging anatomy of the canine cervical region, the dorsal approach was thought to be safer [14]. It has a lower complication rate in terms of iatrogenic injury to important anatomical structures such as the trachea, esophagus, vessels, and nerves. To reduce the risk of the ventral approach, the para-median ventral approach was promoted and used in our study [20]. Drilling the transverse hole in the axis was a procedure with a potential risk of damaging the spinal nerves and vessels in this area. To analyze and reduce this risk, we performed an anatomical evaluation of the lateral approach to C2 in collaboration with the division of the veterinary anatomy. The lateral approach to the cranial part of C2 was safe, and there were no major vessels (such as the carotid artery and its branches) in the surgical field. The C2 nerve root and its branches were clearly visualized during the anatomical evaluation. Therefore, the transverse drill hole in the axis could be safely positioned by gently retracting the lateral muscle mass adjacent to the C2 vertebral body dorsally with a small forceps, as the branches of C2 are more dorsal than the target position of the drill bit. A drill sleeve was used for additional safety and control. The lateral approach for drill hole positioning reduced dorsal and ventral deviation by allowing the drill bit to be placed in the correct position under direct visualization without the ventral musculature in the way. Theoretically, the nerve roots at C2 could be injured if the drill bit was not positioned correctly during surgery. None of our cases appeared to have resulted in nerve root damage. Even if a branch of the C2 nerve root was accidentally damaged when drilling the transverse hole in the axis, no significant clinical signs would be expected.

The main problem associated with the use of the augmentation technique was the correct positioning of the wire/suture. In the first clinical cases, we experienced serious complications (postoperative dysphagia and respiratory dysfunction). In one fatal case, possibly due to excessive manipulation to acquire the transvertebral wire through the hole. Iatrogenic compression of the proximal esophagus and larynx may have led to the clinical signs observed. With increased experience and replacement of the wire with a stronger polypropylene filament that is easier to place and manipulate, these initial complications were eliminated.

To achieve long-term stability of the atlantoaxial joint, atlantoaxial fusion is required. Until bone fusion or fibrosis is achieved, the implants must retain and absorb all forces applied to the atlantoaxial joint. Only ventral stabilization of the atlantoaxial joint can provide adequate long-term fusion, as dorsal approaches leave the articular surfaces intact [2,8,13,22]. To increase the likelihood of fusion and decrease the time to fibrosis, the fixated region should be as stable as possible. If fibrotic tissue formation is delayed, there is a risk of reluxation during this period, which must be avoided. Therefore, the period of fibrotic tissue formation should be kept as short as possible, which can be achieved by stable fixation with reduced range of motion. In a biomechanical study, the new augmented ventral fixation technique showed a reduced range of motion compared to intact and transected ligamentous spines and compared to the dorsal clamp technique [13].

In general, ventral fixation is less prone to failure over time than dorsal fixation [2,3]. Several studies evaluating dorsal fixation have shown a risk of spinal cord injury and an increased risk of implant failure and reluxation/fracture of the C1/C2 segment. Thus, the risk/benefit balance has been debated, and some authors have even questioned the safety of the clinical use of dorsal fixation of the atlantoaxial joint [2,3]. Several ventral fixation techniques have been developed. The main problem affecting all ventral techniques is that the anchoring implants are placed in a ventrodorsal direction, which means that the bony purchase of the implant will be a lee of 2–3 mm in small-breed dogs (height of the axis body). Ventral fixation with PMMA and various implants, such as pins and screws, has the advantage of allowing multipoint fixation. Fixation of a spinal segment with multiple fixation points increases stability and reduces the risk of implant failure by spreading the force over multiple implants. As patients presenting with AAI are often small, immature dogs, fixation with PMMA adds significant weight to the cervical region relative to the patient’s weight. In addition, the volume of PMMA can be difficult to incorporate into the ventral soft tissues. PMMA can also cause thermal damage to the surrounding soft tissue [3,17,23]. As the cervical region is a very delicate anatomical area in toy breed dogs, important structures may be damaged. Complications such as infection and necrosis have also been described [3,17,23].

Ventral transarticular pin or screw fixation distributes force to only two pins, which can lead to implant loosening or fracture over time [2,7]. Due to that, the new augmentation technique could be especially beneficial in patients with transarticular pin or screw fixation. To increase the stability of ventral transarticular fixation, a transverse wire/polypropylene filament was added and attached to the two screws placed in the alae atlantis. The transverse position of the wire in the axis was chosen because the body of the axis is wider than it is tall (8–9 mm vs. 2–3 mm), thus providing greater bone purchase, which increases stability and reduces the risk of implant failure. Large bone purchase is particularly important in animals with very small and fragile bones, such as the classic AAI patient [3]. In addition, the position of the wire/suture crossing the vertebral body of the axis and running ventrally to the atlas body can prevent dorsal dislocation of the axis during shear loading. In combination with another standard fixation, it may be suggested that the augmentation technique may have a neutralizing function against the shear forces that may be responsible for loosening of the primary fixation. The main idea behind augmented fixation is to support the chosen fixation technique by reducing the shear stress on the fixation during movement. As follow-up diagnostic imaging was only performed in two patients, we were unable to confirm or reject this hypothesis with certainty. A previous biomechanical study showed increased stability compared to intact cervical spines in toy breed dogs [13]. Additional biomechanical studies would be needed to further confirm the hypothesis that augmentation has a neutralizing function. The study had several limitations. Due to the retrospective nature of the study, there was no standardized follow-up protocol. We attempted to reduce this limitation by conducting a telephone follow-up with the owners at least six months after surgery. Furthermore, there was no comparison to a control group to investigate the correlation between fixation technique and clinical outcome. Finally, the lack of long-term diagnostic imaging prevented us from testing our hypothesis that the stability of standard fixation techniques could be increased in clinical situations by using the augmentation technique. Due to the small size of the patients, most owners were reluctant to perform follow-up examinations under anesthesia. This issue needs to be addressed in a future study. 

## 5. Conclusions

In conclusion, a ventral augmented fixation technique may be a safe and reliable technique for stabilizing the atlantoaxial joint in toy breed dogs. The positioning of the implants is technically challenging, but the complication rate seems to be low with experience and the choice of suture instead of wire. The clinical results observed were good to excellent and similar to those in previous studies, and further prospective diagnostic imaging studies are needed to confirm the potential clinical biomechanical advantage associated with the use of the augmentation technique. Due to the lack of long-term imaging controls in most patients, we could not confirm that the augmentation technique would prevent or reduce the loosening of the standard implants used for AAI stabilization.

## Figures and Tables

**Table 1:** Detailed information to all 11 dogs participating in the study.

Breeds	Gender	Age	Body Weight	Duration of Clinical Signs until Presentation	Clinical Signs	Surgical Technique	Complications	Clinical Signs after Hospitalization
Havanese	female	16 months	5.2 kg	2 weeks	Ambulatory tetraparetic	Bilateral plating and augmentation technique	Dysphagia and dyspnea post-operatively	Ambulatory with slight tetraparesis
Chihuahua	female	11 months	0.9 kg	10 days	Ambulatory tetraparetic	Transarticular pins and augmentation technique	Dysphagia and dyspnea post-operatively. Death	
Miniature Spitz	female	12 months	2.6 kg	3 weeks	Ambulatory tetraparetic	Transarticular screws and augmentation technique	Dysphagia and dyspnea post-operatively	Ambulatory with slight tetraparesis
Yorkshire Terrier	female	16 months	2.6 kg	2 weeks	Ambulatory tetraparetic	Transarticular screws and augmentation technique	None	Ambulatory with slight tetraparesis
Chihuahua	male	14 months	1.8 kg	2 weeks	Ambulatory tetraparetic	Transarticular pins and augmentation technique	None	Ambulatory with slight tetraparesis
Mix breed dog	female	12 months	11 kg	4 days	Tetraparetic non ambulatory	Bilateral plating and augmentation technique	None	Tetraparetic non ambulatory
Chihuahua	female	15 months	2.1 kg	2 weeks	Pain	Transarticular pins and augmentation technique	None	Ambulatory with slight tetraparesis
Bolonka Zwetka	male	14 months	3.1 kg	10 days	Ambulatory tetraparetic	Bilateral plating and augmentation technique	None	Ambulatory with slight tetraparesis
Miniature Spitz	male	11 months	2.9 kg	7 days	Ambulatory tetraparetic	Bilateral plating and augmentation technique	None	Ambulatory with slight tetraparesis
Mix breed dog	female	6 months	5.5 kg	3 days	Tetraparetic non ambulatory	Bilateral plating and augmentation technique	None	Tetraparetic non ambulatory
Border collie	female	3 months	7 kg	3 days	Tetraplegic with preserved deep pain	Bilateral plating and augmentation technique	None	Ambulatory with slight tetraparesis

## Data Availability

No new data were created or analyzed in this study. Data sharing is not applicable to this article.
